# Comparisons of Ethanol Extracts of Chinese Propolis (Poplar Type) and Poplar Gums Based on the Antioxidant Activities and Molecular Mechanism

**DOI:** 10.1155/2015/307594

**Published:** 2015-02-23

**Authors:** Jianglin Zhang, Xueping Cao, Shun Ping, Kai Wang, Jinhu Shi, Cuiping Zhang, Huoqing Zheng, Fuliang Hu

**Affiliations:** ^1^College of Animal Science, Zhejiang University, Hangzhou 310058, China; ^2^Husbandry and Veterinary Technical Popularization Center of Zhejiang Province, Hangzhou 310020, China

## Abstract

The biological activities of propolis are varied from plant sources and the prominent antioxidant effects of Chinese propolis (poplar type) have been extensively reported. Oxidative stress is associated with inflammation and induces many diseases. In the study, to evaluate antioxidant capacities and clarify the underlying molecular mechanisms of ethanol extracts of Chinese propolis (EECP) and ethanol extracts of poplar gums (EEPG), we analyzed their compositions by HPLC, evaluating their free radical scavenging activities and reducing power by chemical analysis methods. Moreover, we studied the roles of EECP and EEPG on the elimination of ROS and expressions of antioxidant genes (HO-1, TrxR1, GCLM, and GCLC) in RAW264.7 cells. We further investigated the effects of MAPKs on the antioxidant genes expression by specific inhibitors. The nucleus translocation effects of Nrf2 were also measured by confocal microscopy analysis. The results indicated that EECP had higher TPC and FDC values but regarding TFC values were not significant. EECP also possessed more contents of 11 compounds than EEPG. Both phytochemical analysis and cell experiment reflected that EECP exerted stronger antioxidant activities than EEPG. EECP and EEPG enhanced endogenous antioxidant defenses by eliminating reactive oxygen species directly and activating Erk-Nrf2-HO1, GCLM, and TrxR1 signal pathways.

## 1. Introduction

Poplar-type propolis is a resinous substance collected by honey bees from buds of poplar trees. Poplar-type propolis has been studied extensively with broad spectrum biological and pharmacological properties, such as antioxidant [1, 2], anti-inflammatory [3], antiproliferative [4], anticardiovascular diseases [5], antidiabetes [6], and hepatoprotective [7] activities. A large number of biological activities of propolis are based on its complex chemical compositions [8], which are mainly dependent on the plant sources. The previous study reveals that poplar bud (*Populus nigra*) extract contains kinds of phenolic compositions and displays good antioxidant property [9]. The study further shows that poplar propolis displays lower minimum inhibitory concentration (MIC) and minimum bactericidal concentration (MBC) values than poplar buds, which confirms that poplar propolis possesses stronger antimicrobial activity than poplar buds [10]. Moreover, there are growing evidences showing that propolis in market has been partly mixed with ethanol extracts of poplar gums (EEPG), which are extracted from poplar buds. The previous studies have distinguished propolis from poplar gums based on its chemical compositions [11, 12]. Antioxidant property functions as a basic activity of propolis, and there are a large body of reports on it in every year. However, the relationship between Chinese propolis and poplar buds based on antioxidant capacities and their antioxidant molecular mechanisms is still unclear.

Oxidative stress is evoked by increasing intracellular oxidants or reactive oxygen species (ROS), which broke the balance of cellular redox system [13]. Expressions of phase II detoxification and antioxidant enzymes genes are controlled by a cisacting regulatory element termed the antioxidant response element (ARE), which contains genes that are regulated by the nuclear factor erythroid 2-related factor 2 (Nrf2) [14], a member of the Cap“n”Collar/basic-leucine zipper family of transcription factors. Under normal conditions, Nrf-2 is suppressed by Kelch-like ECH-associated protein 1 (Keap1) in the cytoplasm. After activation, Nrf-2 dissociates from Keap1 and transferred into the nucleus to activate the translation of antioxidant genes and phase II detoxification genes [15], such as HO-1 [16], NQO1 [16], and GCLM [14].

As the most abundant cellular thiol, reduced glutathione (GSH) can eliminate electrophiles and ROS efficiently that are generated during chemical metabolism within cells. Glutamate-cysteine ligase (GCL) consists of catalytic (GCLC) and modifier (GCLM) subunits, which is a rate-limiting enzyme of GSH synthesis. Under physiological circumstances, both subunits are required for the synthesis of GSH [14]. HO-1, functions as a phase II enzyme, has also been enormously investigated and the results indicate that HO-1 plays a key role in cellular defense mechanism against oxidative insults [17]. On the other hand, thioredoxin (Trx), members of an evolutionarily conserved family of redox-active proteins, regulates the state of cellular reduction/oxidation and cellular proliferation [18]. Thioredoxin reductase 1 (TrxR1), a selenoenzyme in thioredoxin-system, provides protection effects against oxidative stress [19].

Previously, we have shown that Chinese propolis (poplar type) increased the levels of antioxidant enzymes to prevent hepatorenal injury in vivo [20]. Moreover, the study also confirms that Chinese propolis and poplar gums possess strong potential anti-inflammatory effects in RAW264.7 cells and animal models [21]. Evidence has shown that redox-dependent mechanisms can induce inflammation [22]. Moreover, the study confirms that atherosclerotic lesion is amplified by mitochondrial oxidative stress in lesional macrophages via promoting NF-*κ*B-mediated entry of monocytes and other inflammatory processes [23]. Therefore, RAW264.7 cells were used as a cell model to have a comparison study of the antioxidant activities between poplar-type propolis and poplar buds and further study the possible antioxidant mechanisms. We measured the total phenolic acids content (TPC), total flavonoids content (TFC), and flavanone and dihydroflavonol content (FDC) of ethanol extracts of Chinese propolis (EECP) and ethanol extracts of poplar gums (EEPG), and the main flavonoids and phenolic acids were also determined by High performance liquid chromatography (HPLC). Phytochemical indexes (ABTS, DPPH, ORAC, SRSA, and reducing power) were used to determine the antioxidant activities of EECP and EEPG. We next examined the effects of EECP and EEPG on the inhibition and elimination of ROS produced by H_2_O_2_ in RAW264.7 cells. Furthermore, the mRNA levels and protein contents of antioxidant genes (HO-1, GCLM, and TrxR1) were determined by qRT-PCR and western blot to study antioxidant abilities of EECP and EEPG. To deeply clarify the potential antioxidant signal pathways of EECP and EEPG, the protein contents of JNK/p-JNK, p38/p-p38, Erk/p-Erk, and Akt/p-Akt were also measured with or without inhibitors, and the nucleus translation of Nrf2 were detected by laser scanning confocal microscope analysis. To our knowledge, it is the first time to utilize poplar-type propolis and poplar gums to evaluate the antioxidants and clarify the possible antioxidant mechanisms.

## 2. Materials and methods

### 2.1. Materials

DPPH, ABTS, Trolox, pinocembrin, gallic acid, quercetin, and the standards used in HPLC analysis were purchased from Sigma (St. Louis, MO, USA). Antibodies against HO-1 (lot#:YJ071709CS, catalog#:1922-S, monoclonal), GCLM (catalog#:5529-1, monoclonal), TrxR1 (lot#:YI012101C, catalog#:3928-1, monoclonal), *β*-tubulin (lot#:YH082302D, catalog#:1879-1, monoclonal), p38 (lot#:YF120305C, catalog#:1544-1, monoclonal), Erk1 Phospho/Erk2 Phospho (lot#:YH072803C, catalog#:2219-1, monoclonal), JNK1 phospho/JNK2 phospho/JNK3 phospho (lot#:YK031401CS, catalog#:3893-S, monoclonal), JNK1 (lot#:YH081203, catalog#:3496-S, monoclonal), AKt1 (lot#:YJ051502DS, catalog#:1081-1, monoclonal), and AKt1 phospho (lot#:YH032209C, catalog#:2118-1, monoclonal) were purchased from Epitomics (Burlingame, CA, USA). Antibody against Phospho-p38 (Thr180/Tyr182) (lot#:4, catalog#:45119, monoclonal) was purchased from Cell Signaling technology (Danvers, MA, USA) and anti-Nrf2 (ab31163, polyclonal) antibody was purchased from abcam (Cambridge, Massachusetts, USA). SB203580, SP600125, LY294002, and PD98059 were obtained from Selleckchem (Houston, TX, USA) and the final concentrations used in the experiment were 30 *μ*M, 20 *μ*M, 20 *μ*M, and 20 *μ*M, separately. Other analytical grade chemicals were purchased from Sangon Biotechnology Co. Ltd. (Shanghai, China).

### 2.2. Preparation of Ethanol Extract of Raw Chinese Propolis and Poplar Gums

The Chinese raw propolis was obtained from colonies of* Apis mellifera *in Shandong, China, extracted according to the modified method [3]. In brief, the propolis was extracted with 95% ethanol for three times (200 mL, 150 mL, and 150 mL). The raw propolis ethanol solution was sonicated for 3 h at 40°C. Then, all the solutions were filtered with Whatman number 4 filter papers, collected the residues, and extracted it with 95% ethanol again. Thereafter, the residues were collected and weighed after being dried at 50°C in oven, and then, the propolis yield was calculated according to the method (GB/T 24283-2009). All filter liquors were collected together and filtered to remove wax after being stored at 4°C for a night. Then, all the supernatants were evaporated and dried to a constant weight. The ethanol extract of poplar-type propolis (EECP) was stored at −20°C. On the other hand, ethanol extracts of poplar gums (EEPG) were purchased from the Senlei Plant processing company (Changge, Henan, China), which were extracted from poplar buds (from* Populus *×* canadensis*) by industrial method. A voucher specimen (number 130520) has been deposited at college of Animal Sciences, Zhejiang University. Before the experiment, ethanol extract of poplar-type propolis and poplar gums were redissolved in ethanol and filtered with a 0.22 *μ*m syringe filter. The final concentration of ethanol in the cell culture medium did not exceed 0.1% (v/v) and our preliminary studies had confirmed that this ethanol concentration would not affect following cell experiments.

### 2.3. Total Phenolic Contents (TPC)

The TPC was measured by the modified Folin-Ciocalten method [24]. In brief, 450 *μ*L distilled water was mixed with 10 *μ*L sample and vortexed the solutions for 3 min followed by adding with 10 *μ*L Folin-Ciocalten reagent and then incubated with 30 *μ*L 2% NaCO_3_ at room temperature for 3 h. At last, 200 *μ*L solutions was injected into 96-well plate and measured at 760 nm. In the experiment, different concentrations of gallic acid (150 *μ*g/mL, 200 *μ*g/mL, 250 *μ*g/mL, 300 *μ*g/mL, 350 *μ*g/mL, and 400 *μ*g/mL) were used as a standard and the results were expressed as gallic acid equivalent (GAE) per gram samples.

### 2.4. Total Flavonoids Contents (TFC)

The TFC was determined by the modified method [24]. The reaction solutions were consisted of 150 *μ*L 2% AlCl_3_ and 150 *μ*L ethanol extracts of propolis. The absorbance was measured at 435 nm after incubated at room temperature for 15 min. In the experiment, concentrations of rutin (40 *μ*g/mL~150 *μ*g/mL) were used as a standard to determine TFC in EECP and EEPG. The results were expressed as milligrams rutin equivalent (RE) per gram samples.

### 2.5. Flavanone and Dihydroflavonol Contents (FDC)

The FDC was determined by the method with minor some modifications [25]. Briefly, DNP solutions were prepared by 100 *μ*L 96% sulphuric acid dissolved with 5 mg DNP (2,4-dinitrophenylhydrazine) and diluted to 100 mL with methanol. The reactions consisted of 40 *μ*L sample solutions and 80 *μ*L DNP solutions, and the solutions were incubated at 50°C for 50 min and then cooled and diluted with 280 *μ*L 10% KOH-methanol solutions. Finally, 20 *μ*L reactions were diluted to 1 mL with methanol. The absorbance was determined at 486 nm, and concentrations of pinocembrin (50 *μ*g/mL, 300 *μ*g/mL, 500 *μ*g/mL, 900 *μ*g/mL, 1200 *μ*g/mL, and 1500 *μ*g/mL) were used as a standard to measure FDC in EECP and EEPG. The results were expressed as pinocembrin equivalent (PE) per gram samples.

### 2.6. Determination of 11 Compounds of EECP and EEPG by HPLC Analysis

To separate and determinate the concentrations of 11 compounds in EECP and EEPG, all samples were filtered by 0.22 *μ*m membrane filters and injected 5 *μ*L in Agilent HPLC system, equipped with a vacuum degasser G1322A, a quaternary pump G1311A, an autosampler G1329A, a programmable variable wavelength detector (VWD) G1314B, and a thermostated column compartment G1316A. The conditions of separation are as follows: the temperature of Agilent Eclipse XDB-C18 column (4.6 mm × 150 mm, 5 *μ*m) is 30°C; the mobile phases were performed at a rate of 1.0 mL/min, including (C) acetonitrile and (D) 0.4% acetic acid and gradient elution: 0–40 min, 5–25% (B); 40–45 min, 25–35% (B); 55–60 min, 35–40% (B); 80–90 min, 40–5% (B); 90–100 min, 5% (B). The results were detected at 280 nm and expressed as mean ± SD (*n* = 3).

### 2.7. Evaluation of Free Radical Scavenging Activities and Reducing Power 

#### 2.7.1. ABTS Cation Radical Scavenging Activity (ABTS)

The ABTS cation radical scavenging activity was determined according to the modified method [24]. In brief, 100 *μ*L ABTS working solution (incubated 7.5 mL 7 mM ABTS solution with 132 *μ*L 140 mM potassium persulfate water solution in dark for 16 h and the absorbance of the solution was diluted to 0.7 to get final working solution) was incubated with 50 *μ*L sample in the 96-well plate. The absorbance of the reaction solutions was read at 734 nm after being incubated for 10 min in dark. The results were expressed as IC50 (*μ*g/mL).

#### 2.7.2. DPPH Radical Scavenging Activity (DPPH)

The hydrogen donating activity was evaluated by direct hydrogen donation to the DPPH radical, according to the previous report with minor modification [24]. The reaction solution was consisted with sample and DPPH solutions (1 : 1) and got 100 *μ*L reactions in each well in the 96-well plate to be incubated at room temperature for 30 min in the dark, the absorbance was detected at 517 nm, and the results were expressed as IC50 (*μ*g/mL).

#### 2.7.3. Superoxide Anion Radical Scavenging Activity (SRSA)

The reaction system was modified according to the method [26]. 20 *μ*L sample (propolis or ethanol) and 300 *μ*L 150 *μ*mol/L NBT were mixed with the 300 *μ*L 468 *μ*mol/L NADH. Then, 300 *μ*L 60 *μ*mol/L PMS was added into the mixture and incubated at room temperature for 10 min, and the reaction mixture was transferred into the 96-well plate (200 *μ*L/well and repeats). The absorbance was read at 560 nm. The superoxide anion radical scavenging activities were expressed as trolox equivalent (mmol) per gram of propolis.

#### 2.7.4. Oxygen Radical Absorbance Capacity (ORAC)

The ORAC values were determined by M5 according to the method [2]. Fluorescence (225 *μ*L per well, 8.163 ∗ 10^−8^ mol/L) was added into 96-well black polystyrene plate (Costar), followed by adding sample, blank, or trolox (30 *μ*L per well) and incubated at 37°C for 20 min. AAPH (25 *μ*L, 0.36 M) was added to the mixture and measured every minute for 2 h immediately (excitation wavelength 485 nm and emission wavelength 535 nm). Different concentrations of trolox were used as a standard (12.5 *μ*g/mL, 25 *μ*g/mL, 35 *μ*g/mL, 50 *μ*g/mL, and 75 *μ*g/mL) to measure ORAC value of EECP and EEPG. The results were expressed as the trolox equivalent (mmol) per gram of propolis.

#### 2.7.5. Reducing Power Measurement (RP)

The power of reducing ferric ions was measured by the modified method [27]. 125 *μ*L sample was complemented with 312.5 *μ*L phosphate buffer (0.2 M, pH6.6) and 312.5 *μ*L 1% potassium ferrocyanate. The mixture was preheated at 50°C for 20 min and 312.5 *μ*L 10% trichloroacetic acid was added into the mixture. Then, the mixture was centrifuged at 2000 r/min for 10 min. The supernatants (1 mL) coupled with 312.5 *μ*L distilled water and 62.5 *μ*L 0.1% ferric chloride. 400 *μ*L reaction mixture was pipetted into 96-well plate (200 *μ*L per well), the absorbance was measured at 700 nm, and Trolox was used as the reference sample. The results were expressed as the trolox equivalent (mmol) per gram of propolis.

### 2.8. Cell Culture and Cell Viability Assay

The murine macrophage RAW264.7 cell line, a gift provide by Professor Zongping Xia (Life Sciences Institute, Zhejiang University, China), was cultured with DMEM (Gibco C0005, USA) mixed with 10% fetal bovine serum (Gibco, Australia) at 37°C and 5% CO_2_ in a humidified incubator. RAW264.7 cells were cultured with Petri dish and passaged with 1 : 3 every day. The toxicity of EECP and EEPG was determined by the CCK-8 assay kit (DOJINDO, Japan) according to manufacturer's instructions. The absorbance was determined by an ELISA reader at 450 nm (Bio-Rad 680, USA).

### 2.9. Determination of ROS Production

Cells were treated with indicated concentrations of sample for 0.5 h and then stimulated with 300 *μ*M H_2_O_2_ for further 13 h. Then, cells were washed for twice with PBS to remove the extracellular reactive oxygen species (ROS) and incubated with new medium coupled with 200 *μ*M DCHF-DA for 30 min. After removing culture medium and being washed with PBS, cells were collected by tyrosine and centrifuged at 2500 r/min for 5 min for twice to remove extracellular compounds. Then, the production of ROS was determined by a BD FACSCalibur.

### 2.10. Quantitative Real-Time Polymerase Chain Reaction Analysis (qRT-PCR)

Cells were treated with certain final concentrations of EECP (1.25 *μ*g/mL, 2.5 *μ*g/mL, and 5 *μ*g/mL) and EEPG (5 *μ*g/mL, 10 *μ*g/mL, and 15 *μ*g/mL) for different time duration (3 h, 6 h, 9 h, 12 h, and 24 h). Then, culture medium was removed and total RNA was isolated with RNApure Total RNA kit (AIdlab, China) and concentrations of total RNA were determined by nanodrop spectrophotometer (ND-2000, NanoDrop Technologies). 1 *μ*g RNA was used to conduct reverse transcription and cDNA was synthesized with primeScript RT Reagent kit (TaKaRa, Japan). Finally, qRT-PCR of the cDNA (total cDNA was diluted with RNA enzyme-free water: 1 : 24, v/v) was performed in a final volume of 7 *μ*L with the Mastercycler ep realplex (Eppendorf, Hamburg, Germany) using a SYBR Premix Ex Tag (TaKaRa, Dalian, China) according to the manufacturer's protocol. The reaction conditions were as follows: 95°C for 30 s, 95°C for 5 s, and 60°C for 30 s, followed by the melting curve analysis at 95°C for 15 s, 50°C for 15 s, and 95°C for 15 s. Electrophoresis was utilized to separate PCR products by 1.5% agarose gel, which were stained by GoldView (SBS Genetech, Beijing, China) and visualized under UV light. All of the oligonucleotide primers were designed and synthesized by Sangon Biotech (Shanghai, China). The sense and antisense sequences of primers were as follows:

 GCLC: sense, 5′-GATGATGCCAACGAGTCTGA-3′; antisense, 5′-GACAGCGGAATGAGGAAGTC-3′;
 GCLM: sense, 5′-CTGACATTGAAGCCCAGGAT-3′; antisense, 5′-GTTCCAGACAACAGCAGGTC-3′;
 TrxR1: sense, 5′-AGGATTTCTGGCTGGTATCG-3′; antisense, 5′-CTCGCTGTTTGTGGATTGAG-3′;
 HO-1: sense, 5′-ACATTGAGCTGTTTGAGGAG-3′; antisense, 5′-TACATGGCATAAATTCCCACTG-3′;
 GAPDH: sense, 5′-GAGAAACCTGCCAAGTATGATGAC-3′; antisense, 5′-TAGCCGTATTCATTGTCATACCAG -3′.

### 2.11. Preparation of Protein and Western Blotting

Cells were treated with certain final concentrations of EECP (1.25 *μ*g/mL, 2.5 *μ*g/mL, and 5 *μ*g/mL) and EEPG (5 *μ*g/mL, 10 *μ*g/mL, and 15 *μ*g/mL) for different time duration (HO-1, GCLM, and TrxR1 protein: 3 h, 6 h, 9 h, 12 h, and 24 h; MAPKs protein: 0.25 h, 0.5 h, 1 h, 2 h, and 4 h). At the harvest time, cells were immediately put on ice and washed with cold PBS. Cytoplasmic proteins were lysed with NP40 mixed with protease inhibitors and phosphatase inhibitors and the lysate were collected after scraped by the cell scrapers (corning, USA), vortexed, and put on the ice for 10 min. After that, the lysate were centrifuged with 16000 r/min for 10 min at 4°C and the supernatants were collected and added with certain volume of Laemmli's sample buffer and boiled at 95°C for 10 min and store at −80°C. The concentration of protein was determined by the BCA protein assay kit (Weiao biotechnology, Shanghai, China) and the sample size for western blot was 50 *μ*g. The proteins were separated by the 10% sodium dodecyl sulfate-polyacrylamide gel electrophoresis (SDS-PAGE). After that, the gels were transferred to a polyvinylidene difluoride (PVDF) membrane (Millipore, USA). The membrane was blocked by 5% nonfat milk for 1 h and incubated with primary antibody for 1 h at room temperature, and the antibodies used in the experiment are diluted for western blot, as follows: HO-1, 1 : 2000; GCLM, 1 : 1000; TrxR1, 1 : 1000; *β*-tubulin, 1 : 1000; secondary antibody, 1 : 10000; p38, 1 : 1000; p-p38, 1 : 1000; Akt1, 1 : 1000; p-Akt1, 1 : 1000; p-Erk, 1 : 1000; JNK, 1 : 2000; p-JNK, 1 : 2000 and Nrf2 was diluted with 1 : 100 for Immunofluorescence. After washed 3 times with Tris-buffered saline Tween 20 (TBST), the membrane was incubated with alkaline phosphatase-conjugated secondary antibody for 1 h. The membrane was washed for another 3 times and developed with the method [3]. Finally, the lanes on the membrane were collected by Perfection V300 Photo (EPSON, Japan).

### 2.12. Immunofluorescence Laser Scanning Confocal Microscope

To determine the location of Nrf2, RAW264.7 cells were washed with 2 times for 5 min and fixed with solutions (methanol : acetone, 1 : 1) for 30 min at room temperature. The cells were washed with 3 times for 15 min, then incubated with PBST (0.5% TRITON, Sangon biotechnology) for 30 min, and followed by incubation with 10% goat serum (Boster biological technology, Wuhan, China) for 30 min at room temperature. The cells were treated with anti-Nrf2 antibody (1 : 100) for a night at 4°C and 37°C for 30 min. After being washed with PBS, the cells were incubated with a secondary Alexa fluor 488-conjugated goat anti-rabbit IgG antibody (1 : 500) at 37°C for 1 h. RAW264.7 cells were washed for 3 times and nucleus of cells were stained with DAPI (Beyotime Institute of Biotechnology, Shanghai, China) and then analyzed by laser scanning confocal microscopy.

### 2.13. Statistical Analysis

Data are expressed as mean ± SD and each data representative of three independent experiments. Statistical analysis (Student's *t*-test or one-way ANOVA using the Student-Newman-Keules method) was performed with SPSS17.0 software to determine significant. Values of (^*^ *P* < 0.05), (^**^ *P* < 0.01) and (^#^ *P* < 0.05) were considered statistically significant.

## 3. Results

### 3.1. Total Phenolic Contents (TPC), Total Flavonoid Contents (TFC), and Flavanone and Dihydroflavonol Contents (FDC) of EECP and EEPG

Many evidences revealed that polyphenol exert good antioxidant activities and propolis might attribute to its abundant polyphenolic compounds. The total phenolic contents, total flavonoid contents, and flavanone and dihydroflavonol contents were measured to compare EECP and EEPG. All data were shown in Table 1. The results showed that both TFC values and FDC values of EECP were significant higher than EEPG but TFC values.

### 3.2. Contents of 11 Compounds in EECP and EEPG

**Figure 1:**
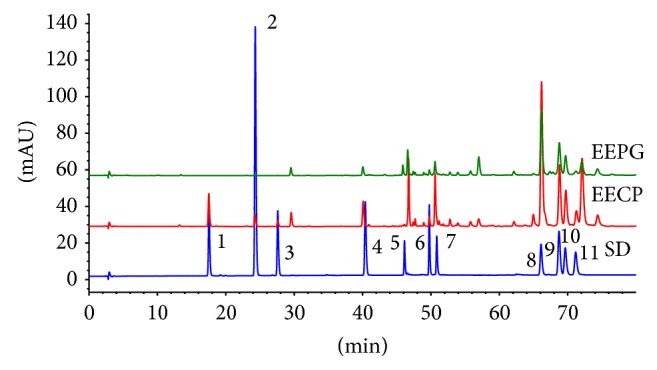
HPLC chromatograms of 11 compounds in EECP and EEPG. 1: caffeic acid; 2:* p*-coumaric acid; 3: ferulic acid; 4: resveratrol; 5: quercetin; 6: apigenin; 7: kaempferol; 8: chrysin; 9: pinocembrin; 10: galangin; 11: caffeic acid phenylethyl ester. SD: standards.

Antioxidant capacities of EECP and EEPG are based on the contents of effective chemical compositions. Accumulating studies have reported that the difference of chemical compositions in propolis and poplar buds is not significant [28, 29]; on the contrary, the previous study has found that salicin can be detected in poplar buds rather than in poplar-type propolis [12]. According to the results of TPC, TFC, and FDC, there is a need to detect the concentrations of some main effective compounds in EECP and EEPG. The concentrations of 11 compounds, which have been reported in poplar buds and poplar-type propolis, were measured by HPLC analysis. The HPLC chromatograms were shown in Figure 1 and their relative concentrations were shown in Table 2. The total contents in EECP were almost more twice than EEPG. Apigenin, chrysin, pinocembrin, galangin, and CAPE were detected in both EECP and EEPG, but resveratrol, quercetin, and kaempferol were not be detected. Caffeic acid,* p*-coumaric acid, and ferulic acid were detected in EECP, but not EEPG.

### 3.3. Free Radical Scavenging Activities and Reducing Power of EECP and EEPG

ROS produced in our body are extremely complicated. Thus, it is difficult to precisely evaluate the total antioxidant capacities with one or two indexes. Thence, four indexes of free radical scavenging capacities and reducing power were chosen to evaluate the total antioxidant activity of EECP and EEPG. The free radical scavenging activities and reducing power were measured by spectrophotometry and data were shown in Table 3. ABTS, SRSA, and ORAC values of EECP were significant higher than that of EEPG. However, EECP and EEPG have the same capacity to eliminate DPPH free radicals. In brief, EECP have stronger free radical scavenging activities and reducing power than EEPG.

### 3.4. Cell Viability of EECP and EEPG in RAW264.7 Cells

**Figure 2:**
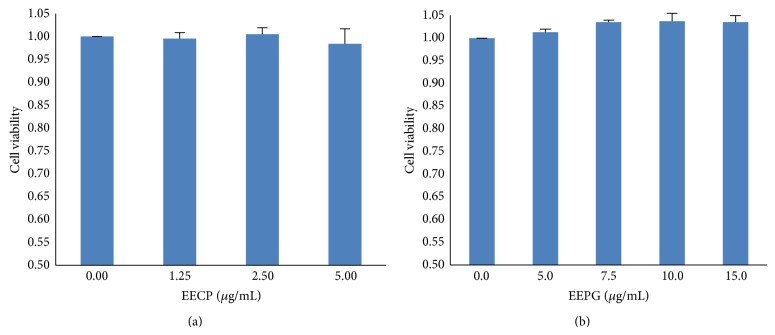
Effects of EECP and EEPG on RAW264.7 cell viability. RAW264.7 cells were treated with indicated concentrations of EECP and EEPG for 24 h, and cell viabilities were determined by CCK-8 assay. The results were the means ± SD (*n* = 3).

The toxicities of EECP and EEPG on RAW264.7 cells were measured by the CCK-8 method according to the protocol. As shown in Figure 2, the results revealed that EECP (5 *μ*g/mL) and EEPG (15 *μ*g/mL) had no toxicity, but higher concentration of EECP and EEPG would be toxic to RAW264.7 cells (data not shown). Thus, the concentrations of subsequent samples were chosen according to the results.

### 3.5. Effect of EECP and EEPG on the Elimination of ROS Stimulated by H_2_O_2_ in RAW264.7 Cells

**Figure 3:**
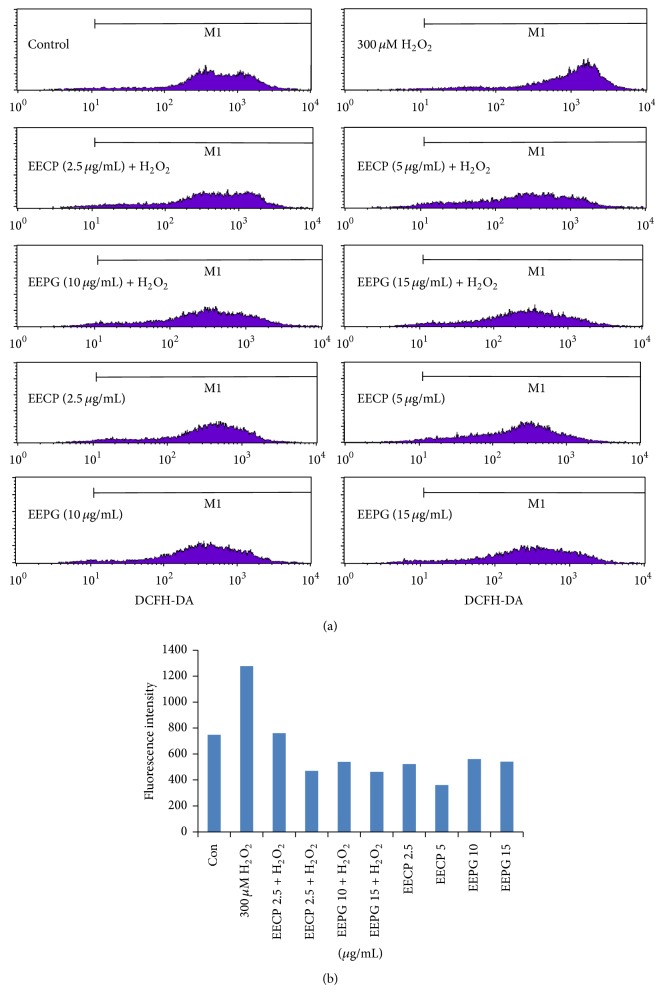
EECP and EEPG reduce the intracellular ROS level in RAW264.7 cells by DCHF-DA assay. RAW264.7 cells were treated with EECP and EEPG, respectively, for 0.5 h, further cultured in the presence or absence of 300 *μ*M H_2_O_2 _for 13 h, and treated with DCHF-DA (200 *μ*M) for 0.5 h. The level of ROS was determined by flow cytometry analysis. (a) A representative result of flow cytometry analysis. (b) Each bar represents the value of fluorescence intensity of each group.

It is not clear whether both EECP and EEPG can effectively scavenge free radicals located in intracellular, although both of them exert good free radical scavenging capacities by chemical analysis. Thus, we investigate the effects of EECP and EEPG on ROS production in the presence or absence of H_2_O_2_. The results of flow cytometry analysis were shown in Figure 3(a), and the data of fluorescence intensity were shown in Figure 3(b). As shown in Figure 3(a), the peak area of H_2_O_2_ treated group, which represented the ROS level in RAW264.7 cells, moved to right in comparison to the control group. However, compared with the H_2_O_2_ treated group, the peak area of the EECP or EEPG treated groups in the presence of H_2_O_2_ moves to the left. The results (Figure 3(b)) directly revealed that H_2_O_2_ significantly induces the production of intracellular ROS in RAW264.7 cells, but the ROS level was decreased significantly by EECP and EEPG, even lower than the normal condition. On the other hand, EECP and EEPG also decreased the ROS level in normal RAW264.7 cells in a dose-dependent manner (Figure 3). Thus, EECP and EEPG could eliminate free radicals effectively against oxidative stress evoked by H_2_O_2_ and inhibit the ROS production in intracellular to enhance the antioxidant capacities of RAW264.7 cells.

### 3.6. Effects of EECP and EEPG on the mRNA Expression of Antioxidant Genes (HO-1, GCLM, GCLC, and TrxR1)

**Figure 4:**
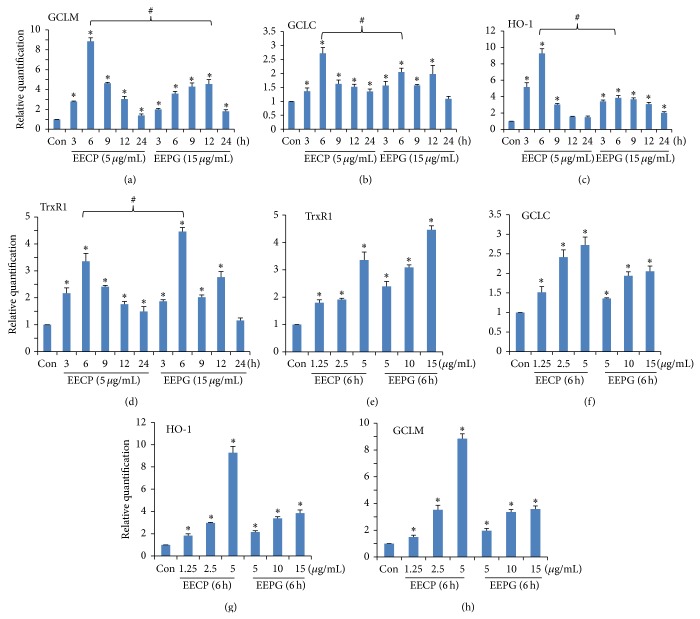
EECP and EEPG stimulated the mRNA expression of antioxidant genes in RAW264.7 cells. ((a)–(d)) To detect the time-course of EECP and EEPG on the mRNA expression of antioxidant genes, cells were treated with EECP (5 *μ*g/mL) or EEPG (15 *μ*g/mL) for indicated lengths of time. ((e)–(h)) RAW264.7 cells were treated with indicated concentrations of EECP and EEPG for 6 h. The expressions of mRNA were measured by qRT-PCR and the results were normalized by GAPDH, which were conducted for three independent experiments and the results were expressed as mean ± SD.

In the study, several related antioxidant genes, including HO-1, GCLM, GCLC, and TrxR1, were chosen to determine the effects of EECP and EEPG. The results were shown in Figure 4. As expected, both EECP and EEPG could dramatically augment the mRNA expression of HO-1, GCLM, and TrxR1 in a time- and dose-dependent manner. However, the mRNA expression of GCLC was not so obvious in comparison to the other genes, although the expression of GCLC was statistically difference (Figures 4(b) and 4(f)). Moreover, at the highest tolerance dose of both samples, EECP stimulated HO-1 and GCLM mRNA expression more efficiently than EEPG, but not TrxR1. More interestingly, the peaks of EECP were appeared at 6 h and a little earlier than EEPG, but EEPG exhibited a more constant effect than EECP. On the other hand, low dose of EECP (1.25 *μ*g/mL, 2.5 *μ*g/mL) gave a slight augmented effect on mRNA expression and high dose of EECP enhanced HO-1 and GCLM mRNA expression dramatically, and the changes did not happen in EEPG (Figures 4(g) and 4(h)).

### 3.7. Effects of EECP and EEPG on the Protein Accumulations of HO-1, GCLM, and TrxR1

**Figure 5:**
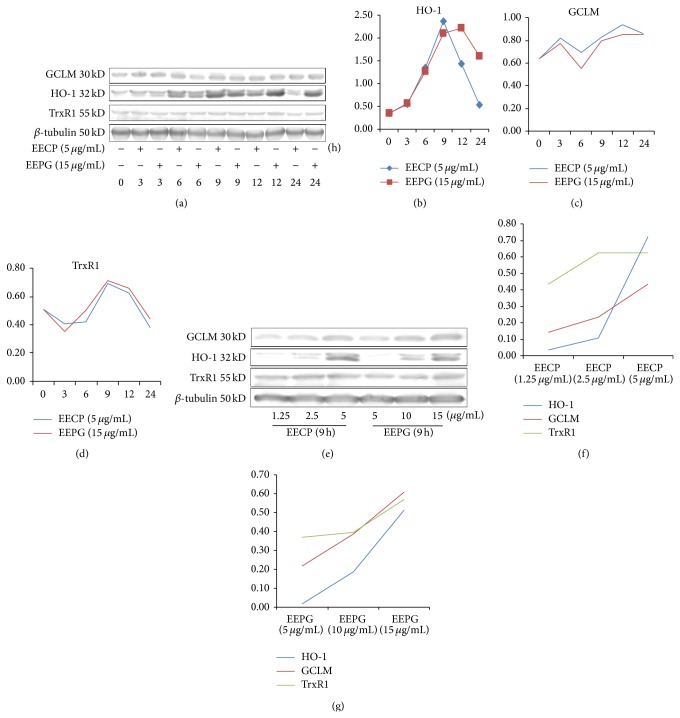
EECP and EEPG activate the expression of antioxidant genes at protein levels in RAW264.7 cells. ((a)–(d)) RAW264.7 cells were cultured in the presence or absence of EECP and EEPG at the indicated lengths time to detect the time-dependent inductions of the HO-1, *γ*-GCLM, and TrxR1 protein. ((e)–(g)) The activation effects of dose-dependent induction of the HO-1, *γ*-GCLM, and TrxR1 protein. RAW264.7 cells were incubated with indicated concentrations of EECP and EEPG for 9 h. All the expressions of the protein were detected by western blot, and the expression of *β*-tubulin protein was used as an internal control. Each value of (b)–(d), (f), and (g) represented the ratio of the density of each stripe of antioxidant genes and the density of each strip of *β*-tubulin protein, respectively. And the values were calculated by image J.

To further study whether EECP and EEPG enhanced the expression of antioxidant genes at protein levers, we determined the protein contents at several time points. The results (Figure 4) indicated that both EECP and EEPG stimulated the expression of HO-1, TrxR1, and GCLM in a time- and dose-dependent manner. However, the stimulation effects of low dose of EECP and EEPG are not so obvious (Figures 5(e)–5(g)) and the results were consistent with the results of mRNA expression. These data also showed that EECP and EEPG had the strongest effects on HO-1, and the activations of GCLM and TrxR1 were not so potent. On the other hand, as the concentrations used were the highest tolerance concentration (Figures 5(a)–5(d)), it was reasonable to compare EECP with EEPG. The results indicated that EECP exerted its functions earlier and stronger than EEPG, but EEPG had more constant effects (Figures 5(a)–5(d)). In total, three antioxidant genes we chosen represent different redox system, and all of them could be stimulated. The results powerful proved that EECP and EEPG had excellent antioxidant capacities.

### 3.8. EECP and EEPG Mediated Antioxidant Genes Expression Mainly through the Phosphorylation of Erk

**Figure 6:**
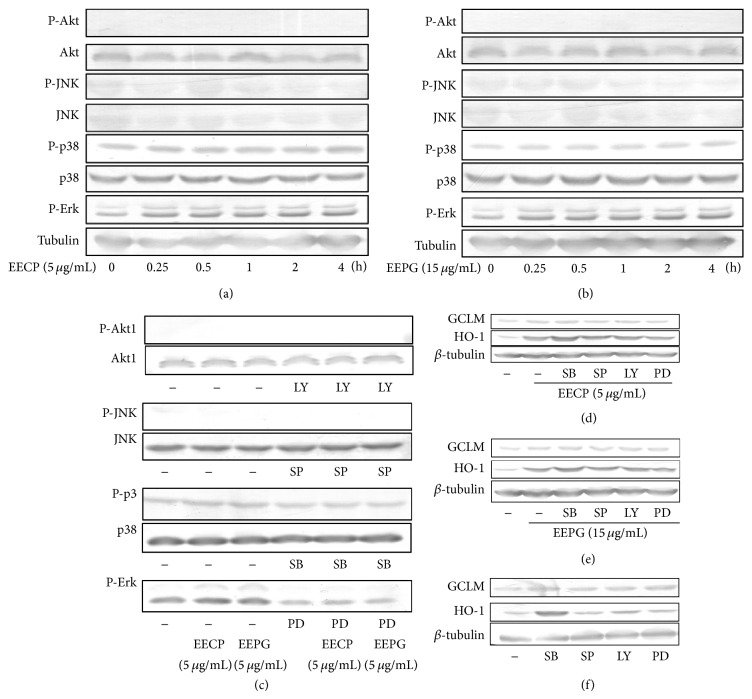
EECP and EEPG mediate antioxidant genes expression mainly through p38/P-p38 and Erk/P-Erk pathways. ((a), (b)) RAW cells were treated with indicated concentrations of EECP and EEPG for following lengths of time, respectively. Then, the cells were harvested with NP40 and cytoplasmic proteins were extracted. Expressions of Akt, phosphorylated Akt, JNK, phosphorylated JNK, p38, phosphorylated p38, and phosphorylated Erk were determined by western blot. (c) RAW cells were pretreated with or without inhibitors (LY294002, 20 *μ*M; SP600125, 20 *μ*M; SB203580, 30 *μ*M; PD98059, 20 *μ*M) for 0.5 h. After that, cells were cultured in the presence or absence of EECP and EEPG on the indicated concentrations for 1 h and the cytoplasmic protein were collected by NP40. Examining the expressions of Akt, phosphorylated Akt, JNK, phosphorylated JNK, p38, phosphorylated p38, and phosphorylated Erk by western blot. ((d)–(f)) RAW264.7 cells were pretreated with or without inhibitors (LY294002, 20 *μ*M; SP600125, 20 *μ*M; SB203580, 30 *μ*M; PD98059, 20 *μ*M) for 0.5 h, followed by culturing with or without EECP or EEPG for 5 h. Then, the medium were removed and cultured RAW264.7 cells with fresh medium for further 4 h. At the harvest time, protein was collected and western blot was used to detect the expression of TrxR1, HO-1, GCLM and *β*-tubulin. *β*-tubulin was used as a protein loading control for each lane.

To test whether the inductions of HO-1, TrxR1, and GCLM were mediated by p38/p-p38, Erk/p-Erk, Akt/p-Akt, or JNK/p-JNK signal pathways, RAW264.7 cells were incubated with EECP (5 *μ*g/mL) and EEPG (15 *μ*g/mL) for indicated length of time. As shown in Figure 6, p-p38 and p-Erk signal pathways were activated by EECP and EEPG, but the effects of EEPG were a little inferior to EECP. The protein levels of p-p38 and p-Erk were elevated at 0.25 h and had some slight increase tendency (Figures 5(a) and 5(b)). However, EECP and EEPG had little effect on Akt/p-Akt and JNK/p-JNK. Previous studies have indicated that HO-1 and GCLM and TrxR1 can be inducted by p38, which could phosphorylate Nrf-2 and accelerate Nrf-2 releasing from Keap1 [30, 31]. Numerous evidences also support the idea that Erk/p-Erk can mediate the induction of antioxidant genes [32, 33]. As a result, we inferred that EECP and EEPG may partly modulate the expression of HO-1, TrxR1, and GCLM via p38/p-p38 and Erk/p-Erk signal pathways.

To confirm our hypothesis, some inhibitors (LY294002, inhibitor of Akt/p-Akt; SP600125, inhibitor of JNK/p-JNK; SB203580, inhibitor of p38/p-p38; PD98059, inhibitor of Erk/p-Erk) were used to inhibit the expression of the correlated proteins. As we expected, Akt/p-Akt and JNK/p-JNK still could not be activated, but the phosphorylation of p38 and Erk was blocked by inhibitors in the presence or absence of EECP and EEPG (Figure 6(c)). We further measured the protein levels of HO-1, GCLM, and TrxR1, after RAW264.7 cells were treated with inhibitors in the presence or absence of EECP and EEPG. Just treated with these inhibitors, the expression of GCLM, TrxR1, and HO-1 were increased by SB203580, but not other inhibitors (Figure 6(f)). Moreover, EECP and EEPG still stimulated the expression of TrxR1, HO-1, and GCLM proteins after pretreated with SB203580 (Figures 6(d) and 6(e)). However, pretreated with PD98059, the stimulation effects of EECP and EEPG on the expression of TrxR1, HO-1, and GCLM were alleviated, while Akt inhibitor and JNK inhibitor did not (Figures 6(d) and 6(e)). As the brightness of TrxR1 lanes was not so high that the lanes could not be clearly scanned by scanner, thus the data of TrxR1 were not shown in Figures 6(d)–6(f). The results indicated that EECP and EEPG induced the expression of HO-1, GCLM, and TrxR1 by activating the phosphorylation of Erk.

### 3.9. Stimulation Effects of EECP and EEPG on the Nucleus Translocation of Nrf2

Many studies have confirmed that herbal extracts can activate the nucleus translocation of Nrf2 to exert antioxidant activities [30, 32]. The previous study also showed that panax notoginseng saponins improved the endogenous antioxidant defenses via Nrf2 pathway to against cell death induced by H_2_O_2_ [34]. In the present study, the results had showed that EECP and EEPG had a marked increase of antioxidant abilities in RAW264.7 cells. Thus, to confirm EECP and EEPG exerted antioxidant functions through the translocation of Nrf2, the location of Nrf2 was determined by laser scanning confocal microscopy. As shown in Figure 6, in the normal conditions, fluorescence was shown as a ring and mainly located in the cytoplasm. Meanwhile, the shape of fluorescence in the treated cells was changed and fluorescence was mainly located in the nucleus. The same situation of nucleus translocation of Nrf2 was also stated in the study [35]. The results indicated that EECP and EEPG stimulated the nucleus translocation of Nrf2.

## 4. Discussion

As we know, numerous studies have confirmed that the chemical compositions of propolis are determined by the plant origin. Propolis contains more than 400 kinds of chemical compositions, but the biologically active substances of poplar-type propolis mainly include flavones, flavanones, cinnamic acids, and their esters [36]. Moreover, fourteen components (chrysin, galangin, pinocembrin, quercetin, ferulic acid, caffeic acid, etc.) are chose as the marker compositions of the poplar buds absolute [37], which might display strong antioxidant capacities [38]. Our results displayed that EECP possessed higher antioxidant activities than EEPG, ascribed to the higher polyphenols contents of EECP than EEPG. The previous studies also confirm that the free radical scavenging capacities and reducing power have obviously correlated with its total phenolic acids, total flavonoids, and monomers [2, 39]. Moreover, the high contents of flavonoids and phenolic acids are main effective constituents of propolis. Although EECP showed higher content of polyphenols than EECP, they also had similar compositions (Table 2). This may the reason why EECP and EEPG stimulated the expression of antioxidant genes via the same signal pathway.

**Figure 7:**
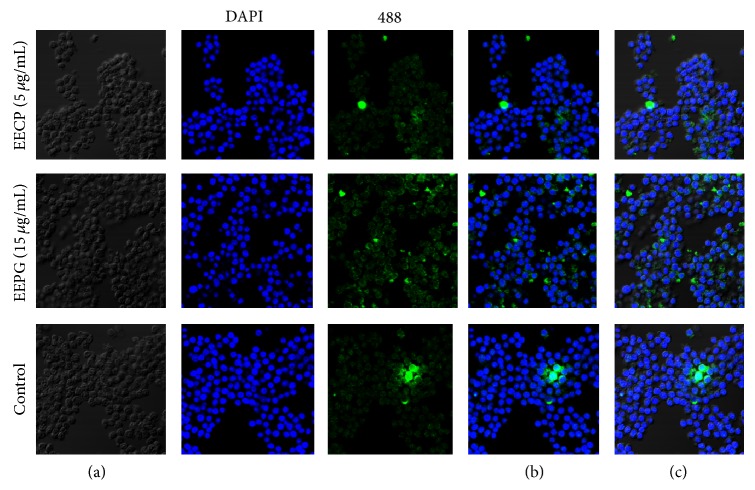
Stimulation effects of EECP and EEPG on the nucleus translocation of Nrf2 in RAW264.7 cells. Nucleus translocation effects of Nrf2 were assessed by laser scanning confocal microscopy. After RAW cells were cultured with indicated concentrations of EECP and EEPG for 4 h, the cells were fixed by methanol-acetone (1 : 1) solutions. Then, the cells were stained with anti-Nrf2 antibody and Alexa Fluor 488-conjugated anti-rabbit IgG antibody and DAPI. (a) The morphology of RAW264.7 cells. (b) The merge of DAPI and Alexa Fluor 488-conjugated anti-rabbit IgG antibody. (c) Merged the pictures of DAPI and Alexa Fluor 488-conjugated anti-rabbit IgG antibody and morphology of RAW264.7 cells to recognize the location of Nrf2 clearly.

Ishige et al. [40] show that flavonoids can eliminate intracellular ROS indirectly by increasing intracellular GSH and decrease the ROS level directly. Reactive oxygen species (ROS) are generated by various physiological and pathological conditions. Accumulating of ROS in intracellular will lead to damages of tissues and cells. Evidence shows that propolis displayed neuroprotection against in vitro and in vivo ischemic neuronal damage induced by oxidative stress [41]. Evidences further reveal that mRNA and protein expression of catalytic subunit (GCLC) and modifier subunit (GCLM) are attributed to the GCL activity, which are recognized as the rate-limiting step in GSH synthesis [22, 23, 42]. HO-1 that functions as a rate limiting enzyme in the breakdown of heme into carbon monoxide, iron, and bilirubin has been extensively studied in recent years [22, 43]. In this study, EECP and EEPG exerted excellent ROS elimination activities (Figure 3) and stimulated the expression of HO-1, GCLM, GCLC, and TrxR1. Hence, EECP and EEPG could improve the redox conditions in cells to against oxidative stress. Additionally, the study confirms [18] the elimination of ROS via Nrf2/HO-1 pathway.  Figure 7 indicated that EECP and EEPG accelerated the Nrf2 located in the cytoplasm transferring into nucleus effectively. Accumulations of Nrf2 in the nucleus bind to the antioxidant response element and upregulate the transcription of antioxidant genes [19], including HO-1, GCLM, GCLC, and TxrR1. Furthermore, previous evidences showed that propolis enhanced the activities of antioxidant enzymes to against the oxidative insults [10, 35].

Although many studies have reported that herbal extracts modulate the expression of HO-1 and GCLM and other antioxidant genes via p38/p-p38, Erk/p-Erk kinases [31, 44], it is the first time to investigate whether propolis via these kinases stimulate the expressions of HO-1, GCLM, and TrxR1. The results indicated that inhibitors of p38 and Erk blocked the expression of p-p38 and p-Erk effectively. Kang et al. and Soo Kim et al. [45, 46] prove that antioxidant genes can be activated via p38/Erk-Nrf2 pathways. As p38 mitogen-activated protein kinase (MAPK) consists of four isoforms: p38*α*, p38*β*, p38*γ*, and p38*δ*. And all of the isoforms are reported to possess a canonical tripeptide sequence (THr-Gly-Tyr) in the activation loop, where there activation is associated with the dual phosphorylation of both threonine (Thr) and tyrosine (Tyr) residues [21]. In the study, the phosphorylation sites of phosphor-p38 antibody contain both threonine (Thr) and tyrosine (Tyr) residues; thus, it is not clear which isoforms of p38 were activated by EECP and EEPG. Furthermore, SB203580 is a specific inhibitor of p38*α* and p38*β* isoforms, and the study also finds that p38*α* and p38*β* isoforms exhibit negative control effect on the induction of antioxidant enzymes [47]. On the contrary, the study [48] shows that SB203580 inhibit the expression of Nrf2 initiated by Diallyl sulfide. Combining the previous studies with our results, we infer that one of p38*α* and p38*β* exerts negative effects on the regulation of antioxidant genes and it will be generated in the normal conditions to inhibit the expression of antioxidant genes, but the other one exhibits positive effects. Of course, the effects of p38*α* and p38*β* may also depend on the cell lines and tissues or conditions. According to the analysis, the activated p38 isoforms by EECP and EEPG may belong to the p38*γ* and p38*δ*. As HO-1 and GCLM are partially modulated via phosphor-Erk kinase, so the stimulation of p38*γ* and p38*δ* may participate in the activation of HO-1 and GCLM. However, the effects of p38*γ* and p38*δ* stimulated by EECP and EEPG on the expression of HO-1 and GCLM genes need to be further studied. Thus, our results indicated that EECP and EEPG stimulated the expression of HO-1, GCLM and TrxR1 via Erk/p-Erk kinase.

Numerous studies have confirmed that HO-1, GCLM, and other antioxidant genes can be activated via Erk kinase/Nrf2 signal pathways [42, 49]. Our results had indicated that EECP and EEPG could accelerate the nucleus translocation of Nrf2 and activate the expression of HO-1, GCLM, and TrxR1 genes by activating Erk kinase. Therefore, we inferred that EECP and EEPG stimulated the expression of HO-1, GCLM, and TrxR1 via Erk kinase/Nrf2 signal pathway.

## 5. Conclusion

In conclusion, EECP and EEPG have similar chemical compositions, but they also display some differences in quality and quantity, which contribute to the differences of antioxidant activities and the same signal pathway. The study indicated that EECP and EEPG possess strong free radical scavenging activities and obviously improve endogenous antioxidant defenses systems. Furthermore, EECP and EEPG exert their potent antioxidant capacities via Erk/Nrf2/GCLM, HO-1, and TrxR1 signal pathway. Meantime, EECP and EEPG can eliminate intracellular ROS directly. Our study gave some insights into studying the function of poplar propolis antioxidant activity on some chronic disease. The result was also helpful to deeply explore health care product of propolis. We will continue to study the antioxidant activity of Poplar propolis in diabetes animal based on the signal pathway stated in the study.

## Figures and Tables

**Table 1:** Total phenolic contents (TPC), total flavonoid contents (TFC), and flavanone and dihydroflavonol contents (FDC) of EECP and EEPG^a^.

Sample^b^	TPC (mg GAE/g)	TFC (mg RE/g)	FDC (mg NE/g)
EECP	192.80^**^ ± 10.85	297.24NS ± 10.32	229.64^**^ ± 7.05
EEPG	121.81 ± 8.83	297.09 ± 10.66	164.14 ± 4.95

^a^Data are shown as the mean ± SD (*n* = 3). ^b^EECP and EEPG represent the ethanol extract of Chinese propolis (poplar type) and ethanol extract of Chinese poplar gum, respectively. NS means not significant; ^*^means significant (*P* < 0.05); ^**^means very significant (*P* < 0.01). GAE: gallic acid equivalent; RE: rutin equivalent; NE: naringenin equivalent.

**Table 2:** Concentration of 11 compounds in EECP and EEPG^a^.

Compounds	Retention Time	EECP	EEPG
	(min)	(g/100 g of extract)	(g/100 g of extract)
Caffeic acid	17.52	0.35 ± 0.00	—
*p*-Coumaric acid	24.32	0.08 ± 0.00	—
Ferulic acid	27.71	0.07 ± 0.00	—
Resveratrol	40.53	—	—
Quercetin	46.24	—	—
Apigenin	49.89	0.12 ± 0.00	0.07 ± 0.00
Kaempferol	50.98	—	—
Chrysin	66.44	2.33 ± 0.06	0.96 ± 0.02
Pinocembrin	69.10	1.22 ± 0.04	0.69 ± 0.01
Galangin	70.04	1.11 ± 0.05	0.65 ± 0.01
CAPE	71.75	0.58 ± 0.04	0.22 ± 0.00

Total		5.85 ± 0.20	2.59 ± 0.05

^a^values are expressed as the mean ± SD (*n* = 3); CAPE: caffeic acid phenylethyl ester; —, not detected.

**Table 3:** Free radical scavenging activities and reducing power of EECP and EEPG.

Sample^a^	DPPH (IC50)	ABTS (IC50)	RP (mmol TE/g)	SRSA (mmol TE/g)	ORAC (mmol TE/g)
EECP	32.35NS ± 2.84	40.5^**^ ± 2.38	2.08^**^ ± 0.08	1.52^**^ ± 0.03	9.25^*^ ± 0.85
EEPG	31.83 ± 2.68	55.4 ± 1.49	1.55 ± 0.05	0.67 ± 0.02	6.43 ± 0.46

^a^Data are showed as the mean ± SD (*n* = 3); statistical analysis were used to compare EECP with EEPG for every index, separately. NS means not significant (*P* > 0.05); ^*^means significant (*P* < 0.05); ^**^means very significant (*P* < 0.01). DPPH and ABTS are expressed as IC50 (*μ*g/mL); FRAP, SRSA, and ORAC are expressed as millimoles Trolox equivalents (TE) per gram of sample.

## Abbreviations

EECP: Ethanol extracts of Chinese propolis
EEPG: Ethanol extracts of poplar gums
HPLC: High-performance liquid chromatography
RP: Reducing power measurement
SRSA: Superoxide radical scavenging activity assay
ORAC: Oxygen radical absorbance capability assay
HO-1: Heme oxygenase -1
GCLC: Glutamate-cysteine ligase catalytic subunit
GCLM: Glutamate-cysteine ligase modifier subunit
TrxR1: Thioredoxin reductases 1
Nrf2: Nuclear factor erythroid 2-related factor 2
Keap1: Kelch-like ECH-associated protein 1
NQO1: NAD(P)H:quinone oxidoreductase 1
p-p38: Phosphor-p38 MAPK
p-Erk: Erk1 phospho/Erk2 phospho
p-JNK: JNK1 phospho/JNK2 phospho/JNK3 phospho
p-Akt: Akt1 phospho
ARE: Antioxidant response element
ROS: Reactive oxygen species.
